# Ethical heuristics for pandemic allocation of ventilators across hospitals

**DOI:** 10.1111/dewb.12315

**Published:** 2021-05-02

**Authors:** César Palacios-González, Jonathan Pugh, Dominic Wilkinson, Julian Savulescu

**Keywords:** COVID-19, decision-making, resource allocation, triage, allocation of ventilators

## Abstract

In response to the COVID-19 pandemic philosophers and governments have proposed scarce resource allocation guidelines. Their purpose is to advise healthcare professionals on how to ethically allocate scarce medical resources. One challenging feature of the pandemic has been the large numbers of patients needing mechanical ventilatory support. Guidelines have paradigmatically focused on the question of what doctors should do if they have fewer ventilators than patients who need respiratory support: which patient should get the ventilator? There is, however, an important higher level allocation problem. Namely, how are we to ethically distribute newly obtained ventilators across hospitals: which *hospital should get the ventilator(s)?* In this paper, we identify a set of principles for allocating newly obtained ventilators across hospitals. We focus particularly on low and middle income countries, who frequently have limited pre-existing intensive care capacity, and have needed to source additional ventilators. We first provide some background. Second, we argue that the main population healthcare aim during the COVID-19 pandemic should be to save the most lives. Next, we assess a series of potential heuristics or principles that could be used to guide allocation: allocation to the most densely populated cities, random allocation, allocation based on the ratio of patients to ICU personnel, prioritisation in terms of intrahospital mortality, prioritisation of younger populations, and prioritisation in terms of population mortality. We conclude by providing a plausible ranking of the principles, while noting a number of epistemological challenges, in terms of how they best further the aim of increasing the probability of saving the most lives.

## 1 Introduction

COVID-19 is a new disease, caused by the SARS-CoV-2 virus, for which currently there is no globally available preventive or curative treatment. The salient clinical manifestations have become clear over the last twelve months. Approximately 80% of people who contract the disease will have mild or moderate symptoms. The remaining 20% will have a serious presentation of the disease and might require hospital care. Of the total number of people infected, 5% develop a severe acute presentation and will require intensive care, and around half of those require mechanical ventilation.^1^ Case-fatality rate (i.e. proportion of deaths among identified confirmed cases) for COVID-19 is not yet well stablished, Rajgor et al. maintain that “the CFR of COVID-19 appears to be lower than that of SARS (9.5%) and Middle East respiratory syndrome (34.4%), but higher than that of influenza (0.1%).”^2^ And more recently Yang Cao et al. have concluded that the “average of country/territory-specific COVID-19 CFR is about 2%-3% worldwide and higher than previously reported at 0.7%-1.3%.”^3^

On March 11, 2019 the WHO declared COVID-19 to be a pandemic.^4^ So far it has spread to 219 countries and territories, and it has killed more than two million people.^5^ It was predicted that if left unchecked COVID-19 could have infected 60% of the global population.^6^ In the UK, for example, the worst case scenario predicted that 80% of the population could have become infected.^7^

The tackling of COVID-19 raises many ethical questions. Among them, the question that has received the most attention is how to ethically allocate scarce medical resources, specifically how to allocate ventilators.^8^ Ventilators are centre stage^9^ on the ethical discussion because international experience showed that even well-resourced healthcare systems had serious problems coping with a large influx of COVID-19 patients requiring them.^10^ In low and middle income countries (LMICs) health systems experienced intense pressure (Box 1).Unlike some other medical resources (e.g. medication, personal protective equipment), ventilators are highly precise instruments which cannot be easily mass produced in a short time. And similar to other medical resources, ventilators are tightly regulated. This means that it does not matter how fast companies produce new ventilators, the demand created because of COVID-19 will outpace global supply in the short and medium term.^11^ All this means that for the time being hospitals around the world will have to work with existing capacity while waiting for new ventilators to trickle down the healthcare system. And this trickle down can also be affected by disruption to global supply chains.

The ethical discussion about how to allocate ventilators during the COVID-19 pandemic has largely focused on how they ought to be allocated between individual patients. Whilst this is an important question, there is an important gap in this discussion; current ethical debates do not consider how governments in LMICs should allocate newly obtained ventilators across hospitals. Here ‘obtained’ must be understood as governments getting hold of more ventilators from manufacturers, or national or international organisations, or from something akin to a national stockpile. The best known example is the US Strategic National Stockpile, whose role is to supplement state and local authorities’ supplies during a public health emergency, and it includes ventilators.^19^ Much of the current ethical debate about allocating ventilators follows what we can call *the intra-hospital model assumption of allocation*. This is, people theorise about the allocation of scarce medical resources while assuming: i) that all the available resources to treat certain disease are already in a hospital, ii) that patients arrive to such a hospital, and iii) that after patients arrive to such a hospital the allocation process takes place.

The aim of this paper is to close the aforementioned gap by exploring a series of principles for governments to allocate newly obtained ventilators across hospitals. This issue is of particular importance for low and middle income countries’ governments which – whilst still managing the first/second wave of infection and the early days of the vaccine rollout – are slowly obtaining new medical equipment during the COVID-19 pandemic and are faced with the question of how to ethically distribute ventilators. For example, countries such Bolivia, Mexico, Panama, Honduras, Peru still do not have adequate numbers of ventilators and are constantly acquiring more.^20^ This issue is also important for high income countries, since now they face a second/third wave of infection, which could require from them to allocate newly obtained ventilators across hospitals.

Before moving to the next section we must clarify that our exploration of the topic assumes that hospitals are already working at capacity or very close to capacity, as is the case in many LMICs. Thus, here we will not explore how we should allocate ventilators in advance of hospitals being in such state, nor how we should allocate ventilators in higher income countries.^21^

## 2 Saving the Most Lives

In order to answer the question of how we should allocate newly obtained scarce medical resources across hospitals we first need to be clear about the aim that we are trying to achieve with them. Resource allocation is ethically complex, and there are debates about which factors might be considered in particular cases of prioritisation. However, a widely accepted principle across different ethical theories is that in the face of a shortage of life saving resources we should try to save as many lives as possible.^22^ In support of this view, from a utilitarian position, we might appeal to the dictum attributed to Bentham that ‘everybody to count for one, nobody for more than one’. If everybody counts for one, and we all have the same value, then it seems intuitive to accept that the more people we can save the better. This insight is captured by Derek Parfit when he writes, “Why do we save the larger number? Because we *do* give equal weight to saving each. Each counts for one. That is why more count for more.” ^23^ Non-utilitarians can accept this principle too, but for different reasons. For example, in a widely cited passage dealing with aggregation Frances Kamm has argued that “If we instead toss a coin between one person and any number on the other side, giving each person an equal chance, we would behave no differently than if it were a contest between one and one, where equipoise can be resolved by the coin toss. If the presence of each additional person would make no difference, this seems to deny the equal significance of each person”.^24^

In working out how to best meet this fundamental goal, we must consider the implications of different allocation decisions across time. To illustrate the point in the context of individual allocation decisions, in such contexts we may have to decide whether to allocate a ventilator to patient X for two weeks, or to patients Y and Z for one week each. In thinking about allocation across hospitals, we may have to consider whether to allocate a ventilator to a hospital that is experiencing a surge in cases now, or one that may experience a more serious surge later. Of course, whilst saving lives is the fundamental goal of ventilator provision, there are plausibly other moral considerations that should feature in our allocation decisions. In particular, it might be argued that allocation decisions should also be grounded by principles of equality and fairness, and that they ought not to instantiate morally problematic forms of discrimination.^25^ Whilst we are sympathetic to this claim, we nonetheless believe that what we have identified as the fundamental goal of ventilator provision should take some degree of precedence over other moral considerations in allocation decisions.^26^

When thinking about the allocation of ventilators to individual patients, if we accept that we should use ventilators in order to maximise the numbers of lives saved then it follows that they should be allocated to those patients with the highest chance of surviving if treated, and who will need the ventilators for the least amount of time. For example, if we could allocate a given ventilator to a patient who will need it for 1 month, or we could allocate the same ventilator to 4 patients that will require it for 1 week then we should choose the latter.^27^ However, for reasons that we shall explain later, the application of the save the most lives principle is more complex in the case of allocation across hospitals in LMICs. For the remainder of the paper we will accept that one of our main aims during the COVID-19 pandemic should be to save the most lives, and thus the allocation principle(s) for newly obtained ventilators across hospitals should, perhaps within certain limits, seek to further this aim.

Before moving forward, some might still resist our stance and argue that considerations of equality/fairness should take priority over the number of lives saved. We accept that there is a radical tension between the save the most lives principle and other principles grounded by considerations of equality/fairness, and that in practice any triaging policy has to make a call on how to weigh these two competing stances. Even when here we cannot adequately address this fundamental issue, our discussion in this paper will add further dimensions to this general debate. It will do so by showing that how we choose to operationalise the save the most lives principle in the allocation of ventilators in LMICs has implications for the precise form of equality/fairness that we may be sacrificing. For instance, allocating ventilators to hospitals that have comparatively low mortality ratios might unfairly benefit comparatively well-off communities. We might avoid this outcome by adopting a different principle, but any other principle we outline will introduce different forms of inequality/unfairness. In the end, ranking the principles that we next explore will be guided by how well we think they operationalise the goal of saving the most lives; however, this ranking will also be influenced by views about which sorts of inequality/unfairness we are willing to accept in the name of saving the most lives.

## 3 Populationdensityprinciple

One intuitively appealing approach would be to allocate newly obtained ventilators to hospitals within the most densely populated cities. Let us call this the Population Density Principle.

*Population Density Principle*: Allocate newly obtained ventilators to hospitals in heavily populated areas

Forexample, in Mexico this would potentially mean sending newly arrived ventilators to Mexico City (which has a population of more than 8 million) and then the remainder between five cities with more than 1.3 million people (Ecatepec, Guadalajara, Puebla, Tijuana, and Ciudad Juarez).

Two reasons speak in favour of this principle. First, the most densely populated cities usually have the largest number of hospitals, which comparatively speaking are well equipped in terms of pre-existing resources and personnel (for example large tertiary hospitals). This would potentially increase the chance that any individual ventilator is able to save a life. A ventilator would go to waste, for example, if we allocated it to a hospital where there is not sufficient medical expertise for operating it. Second, allocating ventilators to hospitals within the most densely populated cities has the theoretical advantage that it does not matter to which specific hospital we allocate it. That is because we can transfer a patient from a hospital where there are not enough resources to another hospital with available resources. The hospitals would be geographically proximate. By contrast, if ventilators were allocated to Oaxaca, for example, hospitals are so distant from each other, and the roads so bad, that transfer of critically ill patients between hospitals is likely to be impossible.

Although intuitively appealing, automatically allocating newly obtained scarce medical resources to hospitals within the most densely populated cities is flawed. First, transfer between hospitals is not always straightforward in some of the most densely populated cities. For example, in Mexico City, the city with the best logistics nationwide, the ambulance infrastructure is not there to safely transport acutely ill patients across hospitals. Furthermore, the pandemic communications among hospitals, and of hospitals with the government, are very poor. For example, a patient in Mexico City ended up visiting 7 different hospitals before being admitted to one, because of poor coordination, and this has been a common feature throughout the pandemic.^28^

Second, there are small(er) cities where their hospitals interact efficiently on a regular basis. In the case of Mexico, for example, Guadalajara seems to do better in this regard than Mexico City. This means that they could transfer acutely ill patients from hospitals without resources to hospitals with resources. All the former discussion about transferring patients presupposes that hospitals in the most densely populated cities and those of the small cities are operating at capacity or close to capacity, but there is no (triaged) waiting list for accessing a ventilator. Once all hospitals have (triaged) waiting lists for accessing ventilators the point about transferring patients becomes moot for the most part, other things being equal. Why? Because (triaged) high priority patients in the waiting list should receive the new ventilator(s). However, it is also true that hospitals in small cities could suffer from the same communication and logistical problems as those in densely populated cities. We can thus conclude that when searching for an allocation principle for newly obtained ventilators we should not assume that acutely ill patients can be transferred across hospitals safely.

Third, we require at least one additional principle to determine how many new ventilators should be allocated to each highly populated area/city. For example, how many ventilators should be allocated to Ecatepec, city of 1.6 million people, in contrast with Guadalajara, city of 1.4 million people? Without this additional principle we are left out in the dark about how to allocate the new ventilators. This issue is not per se an objection to the principle, but it shows its limitations.

Finally, and more importantly, from the fact that the most densely populated cities have the largest number of hospitals – and that they are well equipped in terms of resources and personnel – it does not automatically follow that more lives would be saved if they were to receive the newly obtained scarce medical resources first. Because, other things being equal, the ventilators would save the same number of people in a highly densely populated city as in a less densely populated city. A single ventilator would be able to treat, at a time, one patient in Mexico City or one patient in Cancún. And the same is true even if the resources were to be spread across very small cities. We can thus conclude that the population density principle probably would not lead to saving the most lives.^29^

Before moving to the next section, it could be objected that we have not taken into account that the basic reproduction number^30^ is likely to be greater in densely populated cities than in less densely populated cities, and that thus allocating more ventilators to the former ones would save more lives than allocating them to the latter ones. The quick answer to this question is that we are presupposing that hospitals in both types of cities are working at capacity, or close to capacity, and thus that even when they could have different basic reproduction numbers the situation at the hospitals is the same. It is true that after the initial allocation of new ventilators certain cities might better handle the disease, and their hospitals could no longer be working at capacity, or close to capacity. If this were to be so then we might reallocate ventilators from that city to other cities. A second objection is that higher population density cities will have more patients waiting for a ventilator, and higher priority patients on the (triaged) waiting list. However, this will not necessarily be the case. If less densely populated cities have a lower pre-existing intensive care capacity, or have a worse outbreak, they could have more patients waiting for a ventilator (with a better prognosis) than those in the densely populated city.

## 4 Random Allocation Principle

Given that a new ventilator, other things being equal, would save the same number of people in a highly densely populated city than in a less densely populated city then it might seem intuitive to turn to a Random Allocation Principle.

*Random Allocation Principle*: randomly distribute ventilators between hospitals which have exceeded their capacity to treat patients with their existing resources.

One reason why randomness as a moral principle is attractive, according to John Broome, is that “If a good or bad cannot be distributed equally, it sometimes seems a good idea at least to distribute it randomly. Randomness appears to be a way of bringing some fairness into an inherently unfair situation”.^31^ In this particular case, fairness seems to be achieved in that each hospital has the same chance of getting the ventilator(s). And furthermore, this particular principle of allocation easily resists corruption when carried out transparently. One serious political concern about allocation of ventilators across regions and hospitals, is that such distribution might be susceptible to political influence. Politicians elected from a particular city or region might seek preferential distribution to their electorate. For example, Florida, which is a key battleground state during US presidential elections, was perceived as receiving preferential treatment from the former US administration in terms of resource allocation.^32^ A random process of allocation would avoid such influence and avoid concerns about biased allocation.

Suppose that we have one new ventilator and we are faced with two hospitals, which serve the same type of population, that are working at capacity while patients continue to arrive. In this particular case, other things being equal, the just way to allocate the ventilator would be randomly allocate it (e.g. via a lottery). If other things are indeed equal then we should resort to a random allocation principle. However, it is a mistake to assume that the default position when comparing two (or more) hospitals working at capacity is that things are equal. In order to understand why we need to pay attention to how ventilators work.

A ventilator is not like a magical life preserver that every time we throw it at a group of drowning people it catches someone and does not let her go until she is safe at the beach. Ventilators are just one tool, among many, within ICUs that need to be carefully monitored and managed by doctors, nurses and therapists.^33^ When properly utilised, they increase the probability of a patient surviving COVID-19, and other diseases. For example, for a patient with severe respiratory failure they may increase the chance of surviving from 5% to 50%. However, the magnitude of this increase in chance is not fixed. Thus, when deliberating about how to allocate newly obtained ventilators we should consider how their use increases *the probability of saving the most lives*. It might appear that this change of focus from i) saving the most lives to ii) increasing the probability of saving the most lives is merely semantic, but it is not for two reasons. First, because it corrects a conceptual mistake about ventilators. Second, because it allows us to better identify how things can be different in two hospitals working at capacity or close to capacity.

There is evidence that as the ratio of critically ill patients to ICU healthcare personnel increases the probability of patient survival decreases. It does so because clinical attention is spread across several critically ill patients that need to be carefully monitored, among other reasons.^34^ Imagine the two following scenarios: a hospital has six ICU beds and two ICU nurses per working shift, and another hospital also has six ICU beds but three ICU nurses per working shift. This means that we have a patient nurse ratio of 6:2 and 6:3. Allocating a single ventilator randomly between these hospitals means that we could end up in the two following scenarios 7:2 or 7:3. It is obvious that if we want to increase the probability of saving the most lives, other things being equal, then we should prefer the 7:3 scenario; and thus reject a random allocation principle for this case. This leads us to conclude that if things are not equal in terms of hospital equipment, personnel, and number of patients treated then we should first pay attention to how allocating newly obtained ventilators increases or decreases the probability of saving the most lives. We discuss this further in the next section.

## 5 Patient to Icu Personnel Ratio Principle

We have noted that among hospitals working at capacity we should potentially allocate newly obtained ventilators to those where the patient to ICU^35^ personnel ratio is the lowest. Let us call this the Patient to ICU Personnel Ratio Principle.

*Patient to ICU Personnel Ratio Principle*: when hospitals are working at capacity we should allocate newly obtained ventilators to those hospitals where the patient to ICU personnel ratio is the lowest.

Allocating ventilators in this way increases the probability of saving the most lives, when compared against allocating the ventilators to hospitals where the patientto ICU personnel ratio is higher. For example, in Mexico this principle would most probably favour the allocation of new ventilators to the National Institute of Respiratory Diseases. However, it is also worth considering a potential countervailing consideration. When we add a new ventilator to a hospital the probability of survival for one new patient (who would otherwise have missed out on treatment) increases dramatically. But, at the same time, the medical care and probability of survival for patients already in ICU may reduce, because of the reduction in the availability of nursing and medical staff. For example, when a new ventilator arrives an ICU nurse who previously cared for three patients might now have to care for four patients simultaneously. The three existing patients experience a reduction in the amount of time and attention this nurse is able to devote to them. This would potentially translate into an increased incidence of complications and even into increased mortality. An additional point to consider is that COVID-19 contagion among healthcare professionals in LMICs is very high, and this could affect how staffing considerations are dealt with when deciding how to allocate ventilators. In Mexico, for example, in the run up to September 2020 more than 100,000 healthcare professionals had contracted COVID-19, and more that 1,000 healthcare professionals had died from it.^36^

This consideration does not mean that additional ventilators should not be purchased or added to a hospital. A reduction in the care and outcome of all already treated ICU patients (or group of already treated ICU patients being looked after by a specific team) would happen in any hospital working at capacity that receives a new ventilator.^37^ And the overall benefit (in terms of increasing the probability of saving the most lives) is likely to be greater from treating additional patients, even if some existing patients are worse off. Yet, there may be a point where this is no longer true. There may be a threshold in terms of the patient to ICU personnel ratio beyond which additional ventilators do not increase the probability of saving the most lives.^38^

## 6 Intrahospital Mortality Principle

The patient to ICU personnel ratio principle would prioritise hospitals that have a greater capacity (in terms of physical resources and staff) to ventilate additional patients, if they only had extra ventilators. However, even if one hospital would be able to more efficiently ventilate additional patients, that would not necessarily translate into increasing the probability of saving the most lives. There may be other complex factors to do with the skills and expertise of the staff involved that mean that one hospital is much more successful in saving the lives of patients with COVID-19 than another. If there are differences between hospitals in their survival rates for ventilated patients during the pandemic, prioritising those hospitals with lower mortality might increase the probability of saving the most lives. For example, if the mortality in hospital A is 40%, while it is 60% in hospital B, providing an additional ventilator to hospital A would more likely increase the probability of saving the most lives.

*Intrahospital Mortality Principle*: allocate newly obtained ventilators to hospitals with the lowest mortality.

Although this principle appears plausible, it also comes with some challenges. For example, it may be extremely difficult to obtain up to date data during a pandemic about the relative mortality in different hospitals.^39^ Particularly in lower resourced settings that are overwhelmed by critically ill patients there may be no staff or time to collect and collate data. Moreover, data may not be reliable.^40^ For example, throughout the pandemic the Mexican federal government has been accused of underreporting COVID-19 deaths, in order to give the impression that it is under control.^41^

Next, mortality in different hospitals will be affected by the ethical decision-making of that institution. For example, a hospital that attempts to treat in intensive care all patients who would potentially benefit will have a higher mortality rate than another hospital that is highly selective and only ventilates patients with better prognosis, while discharging ‘terminal’ patients for them to die at home or on the wards. This difference in COVID-19 treatment, for example, happens all across Mexico, since doctors have a great deal of discretion in terms of how to treat patients. Also, hospital mortality figures will be influenced by case-mix – by the characteristics of the population served by the hospital. That might benefit hospitals whose patients have fewer poor prognostic factors. Finally, the low mortality of a hospital could indeed be related to its patient to ICU personnel ratio. While theoretically attractive, this principle is pragmatically hard to implement in a reliable way.

## 7 Population Age Principle

One of the distinctive features of the COVID-19 pandemic is its disproportionate impact on older members of the population: the death rate increases with age.^42^ Thus, if we want to improve the probability of saving the most lives then we should allocate newly obtained ventilators to those hospitals that not only have the necessary extra resources and lowest patient to ICU personnel ration, but are also located in places where the population is comparatively younger. Let us call this the Population Age Principle.

*Population Age Principle*: allocate newly obtained ventilators to hospitals serving a younger population.

Suppose that the mean age of the population being served by hospital A is 50 years old whereas the mean age of the population being served by hospital B is 70 years old. Other things being equal (and assuming that both hospitals have exceeded their intensive care capacity), we should allocate the extra ventilator to hospital A. Doing so increases the probability of saving the most lives.^43^ It could be objected that allocating ventilators to hospitals in this way is a form of ageism. The claim might be that we are unjustly discriminating against those populations with the highest number of older people because we consider that their lives are of less value. However, the population age principle is not inherently tied to a specific age, but rather it is responsive to evidence about specific diseases and survival rates. It would mean, for example, that in a different pandemic, that affected younger patients more than older patients (e.g. as occurred in the 1918 influenza pandemic), other things being equal, we would allocate new obtained ventilators to hospitals that are located in cities where the mean age is older.^44^ It is important to highlight that once we had allocated newly obtained ventilators to those particular hospitals internal resource allocation protocols would still take place.^45^

Whereas the population age principle furthers our aim of increasing the probability of saving the most lives there are at least two pragmatic considerations that need to be taken into account before resorting to it. First, it could well be the case that governments in some LMICS do not have up to date data on the age-range of the population that a certain hospital serves during the pandemic. This can be so because the government does not collect or curate the data properly, or because the pandemic causes migration patterns that affect the population that a specific hospital serves. For example, earlier in the year India saw mass internal migration due to lockdown measures.^46^ Second, there could be shielding measures in place to protect the elderly from coming into contact with the virus. For example, a policy that mandates those over a certain age not to leave their homes until a certain date.^47^ Shielding policies, if effective, could have the consequence of lowering the age-range of the population that could most likely come into contact with the virus. Similarly, depending on social distancing policies and their uptake, spread of the virus might be greater amongst the young and lead to a shift in the age of those becoming unwell. For example, data from May 2020 shows that in Mexico infection rates in children and adolescents went up when compared to that of seniors.^48^ This should make us look for another principle.

## 8 General Population Mortality

Since population age is only contingently relevant (i.e. insofar as it predicts the underlying mortality, and the impact of ventilator allocation on survival), an alternative principle would focus more directly on survival.

*General Population Mortality Principle:* allocate newly obtained ventilators to hospitals serving the population with the lowest mortality rate.

Like the intra-hospital mortality principle, this principle would potentially have the greatest and most direct impact on survival. Providing ventilators to cities or regions with a lower mortality rate would lead to more survivors. However, one challenge (also relevant to the intrahospital mortality principle) is that such a principle may exacerbate existing socio-demographic disadvantage. For example, indigenous populations in Mexico have a 70% higher chance of dying from COVID than the rest of the population.^49^ There are indicators that the higher mortality rate is not solely linked to biology, but to social determinants of health. Such populations suffer higher rates of pre-existing poor health, suffer higher rates of malnutrition, are more likely to live in deprived areas, are more likely to live in inadequate and overcrowded conditions, and are more exposed to pollutants, all which could influence mortality from COVID-19.^50^ Therefore, if we were to follow a general population mortality principle, other things being equal, then it is quite possible that the newly obtained ventilators should be allocated away from the hospitals that serve these communities.

Of course, this troublesome implication of following a general population mortality principle in the allocation of ventilators will only arise in certain countries. There are high income countries, for example Japan or Norway, and low and middle income countries, for example Haiti, with highly homogenous ethnic populations. In these countries resorting to the general population mortality principle would not unjustly discriminate against certain ethnic groups.

However, for countries like Mexico, Brazil, and Peru where there are differences in mortality that appear to be related to ethnic background, simply allocating in accordance with a general population mortality principle could entail this unjust form of discrimination. This may give us sufficient reason to disregard the general population mortality principle as a valid principle for allocation. Indeed, it might lead us to adopt allocation principles that not only circumvent the morally problematic forms of discrimination, but which actively seek to redress it. For instance, we could try to reduce health disparities and allocate the newly obtained ventilator(s) to the hospitals that serve or sit within disadvantaged communities.^51^ Here is one clear way in which the value of minimizing mortality comes into conflict with other core values, such as equality. Our purpose in this paper is not to resolve that conflict, but rather to highlight how it arises in the context of allocating ventilators in LMICs. Allocating in a manner that specifically targets disadvantage communities can be understood as one of rectificatory justice towards these communities. This is a morally laudable aim, but we should not be oblivious to the fact that pursuing this aim will require that we accept a greater number of lives lost during the pandemic.

## 9 Conclusion

In this paper we have presented and explored six different principles that could be used for allocating newly obtained ventilators across hospitals. We concluded that the Population Density Principle is found wanting and should not be relied upon. We also concluded that the Random Allocation Principle is inadequate for achieving the aim of increasing the probability of saving the most lives when first allocating new ventilators; but that it should be employed when faced with the task of allocating a ventilator between two hospitals when things are equal between them. Identifying the way in which the remaining four principles should be ranked in order to increase the probability of saving the most lives is difficult for the epistemological (e.g. we do not have adequate data) and other reasons that we have explored (e.g. intrahospital mortality is affected by the ration of patients to ICU personnel). Thus, a first conclusion is that governments and institutions should strive to collect and collate data in the best possible way.

Now, here we offer a plausible ranking of the principles that would further the aim of increasing the probability of saving the most lives. We could first assign new ventilators according to the Intrahospital Mortality Principle, attempting to correct for distorting factors like different case-mix. As we have already discussed, previous ethical decisions and staff numbers play a very important role in which patients are treated and survive. Thus the ratio of patient to ICU personnel could be used as a limiting factor^52^ or a tie breaker between hospitals. If it were to be the case that data regarding intrahospital mortality is unavailable or unreliable, we could then resort to the Patient to ICU Personnel Ratio Principle, and if data was not available then, as a final option, we could move on to the General Population Mortality Principle, i.e. prediction of mortality based on known population factors. In certain scenarios the General Population Mortality Principle has the implication of disfavouring groups that already are affected by health inequalities and structural injustices, for example indigenous communities. It might seem that the best course of action in such circumstances is to allocate the new ventilators to the hospitals serving those communities. Doing so could end up in three different scenarios, depending on the hospitals’ circumstances. First, it could well be the case that a hospital has few material resources but an adequate numbers of personnel. In this scenario we end with the same allocation result as if we were following the Patient to ICU Personnel Ratio Principle. Second, we could have a hospital that is completely stretched out in terms of personnel (or other material resources) and where receiving additional ventilators would not benefit any patient. It is obvious that if this were to be the case then this hospital should not receive the new ventilators (unless it can be assigned additional personnel and other material resources) and they should be sent to another hospital, otherwise the ventilators would go to waste. Finally, there could be cases where the allocation of new ventilators would not further the aim of increasing the probability of saving *the most lives*, but nonetheless would increase the probability of *saving lives*. Let us remember two things: i) these three scenarios only obtain if we act against the General Population Mortality Principle where it disadvantages communities affected by health inequalities and structural injustices, and ii) that once the new ventilators have been allocated internal triage policies would still apply. In conclusion, if we are at all attracted to increasing the probability of saving the most lives, we will at least need to consider these principles in the light of available local information. In this way, practical ethics is context dependent.

## Figures and Tables

**Table 1:** Allocation principles

Principle	Implications
1. Population Density Principle	Allocate newly obtained ventilators to hospitals in heavily populated areas
2. Random Allocation Principle	Randomly distribute ventilators between hospitals who have exceeded their capacity to treat patients with their existing resources
3. Patient to ICU Personnel Ratio Principle	When hospitals are working at capacity we should allocate newly obtained ventilators to those hospitals where the patient to ICU personnel ratio is the lowest.
4. Intrahospital Mortality Principle	Allocate newly obtained ventilators to hospitals with the lowest mortality
5. Population Age Principle	Allocate newly obtained ventilators to hospitals serving a younger population
6. General Population Mortality Principle	Allocate newly obtained ventilators to hospitals serving the population with the lowest mortality rate.

## BOX 1 The Mexican ventilator crisis

On January 28, 2020 the Mexican authorities announced that the public health sector had 6,175 ventilators.^12^ This announcement came as the government made preparations for the arrival of COVID-19 to the country of 126 million people. Among the preparations put in place, the government divided public hospitals into those which would treat COVID-19 patients and those which would not. By mid-March the government announced that the public health system, in addition to the Naval Secretariat and the National Defence Secretariat, had a total of 3,000 ICU beds for treating COVID-19 patients, and of those 400 of them were in Mexico City, city of 8.9 million people.^13^ Interestingly, the Mexican government never publicly said how many ventilators they thought they would need in order to adequately deal with the pandemic, even when they publicly opted for a mitigation strategy rather than an elimination one. However, foreseeing that the amount of available ventilators would not be enough for the country the government set out to buy more. They did so not only because of the comparatively small number of ventilators to population that they had, but also because at least 1,364 of their existing ventilators were broken down.^14^ If these figures are to be believed that meant that by April 2020 the public health sector had ~4,8ll functional ventilators.

In April the Mexican government drew a plan for the staggered purchase of 2,7llnew ventilators: 11 in April, 440 in May, 160 in June, 1020 in July, 1030 in August, and 50 in September.^15^ At present, the total number of new ventilators that the government has received is unknown, due to the lack of transparency. We do know, however, that 561 ventilators have been delivered from the US and ~1050 from China.^16^ At the time of writing public and private hospitals treating COVID-19 patients in Mexico City are already full, and hospitals that originally were not supposed to treat COVID-19 patients are already doing so.^17^ This means that ICUs in Mexico City are already running at capacity, or very close to capacity, and that patients are not only not being admitted to them, but sometimes turned away from hospitals altogether.^18^ What is happening in Mexico City is happening all across the country, since the infection rate keeps rising. This raises the question: where should the newly arriving ventilators be sent?

## Footnotes

1 Guan, W., Ni, Z., Hu, Y., Liang, W., Ou, C., He, J Zhong, N. (2020). Clinical Characteristics of Coronavirus Disease 2019 in China. *New England Journal of Medicine*. https://doi.org/10.1056/NEJMoa2002032

2 Rajgor, D.D., Lee, M.H., Archuleta, S., Bagdasarian, N., & Quek, S.C. (2020). The many estimates of the COVID-19 case fatality rate. *The Lancet Infectious Diseases. 20*(7), 776-777.

3 Cao, Y., Hiyoshi, A., & Montgomery, S. (2020). COVID-19 case-fatality rate and demographic and socioeconomic influencers: worldwide spatial regression analysis based on country-level data. *BMJ Open. 10*(11), eO4356O.

4 WHO. (2020, March 11). WHO Director-General’s opening remarks at the media briefing on COVID-19 - 11 March 2020. Retrieved March 8, 2021, from https://www.who.int/dg/speeches/detail/who-director-general-s-opening-remarks-at-the-media-briefing-on-covid-19---11-march-2020

5 European Centre for Disease Prevention and Control. (2020, August 31). COVID-19 situation update worldwide, as of week4, updated 4 February 2021. Retrieved 9 February 2021, from https://www.ecdc.europa.eu/en/geographical-distribution-2019-ncov-cases

6 Boseley, S. (2020, February 11). Coronavirus ‘could infect 60% of global population if unchecked’. *The Guardian*. Retrieved March 8, 2021, from https://www.theguardian.com/world/2020/feb/11/coronavirus-expert-warns-infection-could-reach-60-of-worlds-population

7 Wilson, L., & Hutchison, C. (2020, March 3). 80% of population could become infected by coronavirus. Retrieved April 28, 2020, from https://www.bbc.co.uk/news/live/uk-scotland-scotland-politics-51698000

8 SIAARTI. (2020, March 6). Clinical Ethics Recommendations for Admission to Intensive Care and for Withdrawing Treatment in Exceptional Conditions of Imbalance Between Needs and Available Resources. Retrieved March 8, 2021, from http://www.siaarti.it/SiteAssets/News/COVID19%20-%20documenti%20SIAARTI/SIAARTI%20-%20Covid-19%20-%20Clinical%20Ethics%20Reccomendations.pdf; Emanuel, E.J., Persad, G., Upshur, R., Thome, B., Parker, M., Glickman, A.,... Phillips, J.P. (2020). Fair Allocation of Scarce Medical Resources in the Time of Covid-19. *New England Journal of Medicine*. https://doi.org/10.1056/NEJMsb2005114; MathenyAntommaria,A.H.,Gibb,T.S., McGuire,A.L., Wolpe, P.R., Wynia, M.K., Applewhite, M.K.,... for a Task Force of the Association of Bioethics Program Directors. (2020). Ventilator Triage Policies During the COVID-19 Pandemic at U.S. Hospitals Associated With Members of the Association of Bioethics Program Directors. *Annals of Internal Medicine*. https://doi.org/10.7326/M20-1738; Maves, R.C., Downar, J., Dichter, J.R., Hick, J.L., Devereaux, A., Geiling, J.A.,... Christian, M.D. (2020). Triage of Scarce Critical Care Resources in COVID-19 An Implementation Guide for Regional Allocation: An Expert Panel Report of the Task Force for Mass Critical Care and the American College of Chest Physicians. *Chest*. https://doi.org/10.1016/j.chest.2020.03.063; Rosenbaum, L. (2020). Facing Covid-19 in Italy — Ethics, Logistics, and Therapeutics on the Epidemic’s Front Line. *New England Journal of Medicine*. 382:1873-1875. https://doi.org/10.1056/NEJMp2005492; Savulescu, J., Cameron, J., £Wilkinson, D. (2020). Equality or utility? Ethics and law of rationing ventilators. *British Journal of Anaesthesia*. https://doi.org/10.1016/j.bja.2020.04.011Truog, R.D., Mitchell, C., & Daley, G.Q. (2020). The Toughest Triage — AllocatingVentilators in a Pandemic. *New England Journal of Medicine*. https://doi.org/10.1056/NEJMp2005689; White, D.B., & Lo, B. (2020). A Frameworkfor RationingVentilators and Critical Care Beds During the COVID-19 Pandemic. *JAMA*, https://doi.org/10.1001/jama.2020.5046; Wilkinson, D. (2020). ICU triage in an impending crisis: uncertainty, pre-emption and preparation. *Journal of Medical Ethics*. medethics-2020-106226. https://doi.org/10.1136/medethics-2020-106226

9 That this question is the most discussed one does not mean that it is the most important one.

10 Healy, M. (2020, April 7). Ventilators for coronavirus patients are in short supply. How scientists might pivot. Retrieved April 28, 2020, from https://www.latimes.com/science/story/2020-04-07/researchers-look-for-ways-to-divert-patients-from-ventilators-as-shortage-looms; Armangue, B., & Wilson, J. (2020, April 2). Agonizing decisions being made in Spain’s virus hot spots. Retrieved April 28, 2020, from https://apnews.com/a6dacb2O42657df4bb36Od9e6fe8O2bf; Rosenbaum (op. cit. note 8)

11 Davies, R., & Rankin, J. (2020, April 3). NHS faces shortfall of ventilators as manufacturers struggle. *The Guardian*. Retrieved March 8, 2021, from https://www.theguardian.com/business/2020/apr/03/nhs-faces-shortfall-of-ventilators-as-manufacturers-struggle-coronavirus

19 US Department of Health & Human Services. (2020, April 3). Strategic National Stockpile. Retrieved April 28, 2020, from https://www.phe.gov/about/sns/Pages/default.aspx

12 Secretaría de Salud. (2020, January 28). El Pulso de la Salud. Retrieved March 8, 2021, from https://www.gob.mx/cms/uploads/attachment/file/529236/CPM_Pulso_de_la_Salud__28ene20.pdf

13 Vega, A., & Daen, A. (2020, March 18). Con pocas camas y bajo número de médicos México enfrenta al COVID-19. *Animal Político*. Retrieved March 8, 2021, from https://www.animalpolitico.com/2020/03/pocas-camas-hospital-baja-proporcion-medicos-enfrentaria-mexico-covid-19/

14 LatinUS. (2020, April 13). El Institute de Salud de AMLO tiene 2,343 ventiladores, pero 58% de ellos no funcionan. *LatinUS*. Retrieved March 8, 2021, from https://latinus.us/2020/04/13/instituto-salud-amlo-ventiladores-no-funcionan/

15 Gobierno de México. (2020, April 6). Programa de Abasto de insumos | COVID 19. Retrieved March 8, 2021, from https://www.gob.mx/cms/uploads/attachment/file/54556l/CPM_Programa_de_Abasto_de_lnsumos__07abr20.pdf.pdf

16 INFOBAE. (2020, August 12). Llegaron a México los últimosventiladores del puente aéreo con EEUU. Retrieved 17 August 2020, from /america/me×ico/2020/08/12/llegaron-a-mexico-los-ultimos-ventiladores-del-puente-aereo-con-eeuu/TV Azteca. (2020, July 11). Por puente aéreo México-China han llegado más de mil ventiladores para Covid-19. Retrieved September 1, 2020, from https://www.tvazteca.com/aztecanoticias/notas/por-puente-aereo-mexico-china-han-llegado-mas-de-mil-ventiladores-para-covid-19INFOBAE. (2020, August 12). Llegaron a México los últimosventiladores del puente aéreo con EEUU. Retrieved 17 August 2020, from /america/me×ico/2020/08/12/llegaron-a-mexico-los-ultimos-ventiladores-del-puente-aereo-con-eeuu/TV Azteca. (2020, July 11). Por puente aéreo México-China han llegado más de mil ventiladores para Covid-19. Retrieved September 1, 2020, from https://www.tvazteca.com/aztecanoticias/notas/por-puente-aereo-mexico-china-han-llegado-mas-de-mil-ventiladores-para-covid-19INFOBAE. (2020, August 12). Llegaron a México los últimosventiladores del puente aéreo con EEUU. Retrieved 17 August 2020, from /america/me×ico/2020/08/12/llegaron-a-mexico-los-ultimos-ventiladores-del-puente-aereo-con-eeuu/TV Azteca. (2020, July 11). Por puente aéreo México-China han llegado más de mil ventiladores para Covid-19. Retrieved September 1, 2020, from https://www.tvazteca.com/aztecanoticias/notas/por-puente-aereo-mexico-china-han-llegado-mas-de-mil-ventiladores-para-covid-19INFOBAE. (2020, August 12). Llegaron a México los últimosventiladores del puente aéreo con EEUU. Retrieved 17 August 2020, from /america/me×ico/2020/08/12/llegaron-a-mexico-los-ultimos-ventiladores-del-puente-aereo-con-eeuu/TV Azteca. (2020, July 11). Por puente aéreo México-China han llegado más de mil ventiladores para Covid-19. Retrieved September 1, 2020, from https://www.tvazteca.com/aztecanoticias/notas/por-puente-aereo-mexico-china-han-llegado-mas-de-mil-ventiladores-para-covid-19

17 Martínez, Á.C. (2020, May 12). Están llenos hospitales Covid en el Valle de México. *La Jornada*. Retrieved from https://www.jornada.com.mx/ultimas/politica/2020/05/12/estan-llenos-hospitales-covid-en-el-valle-de-mexico-8215.html; Animal Político. (2020, May 5). CDMX habilita dos hospitales más para atender a pacientes con COVID. *Animal Político*. Retrieved March 8, 2021, from https://www.animalpolitico.com/2020/05/cdmx-habilita-dos-hospitales-atencion-pacientes-covid19/

18 Reporte Indigo. (2020, May 14). Tras recorrer siete diferentes hospitales, hombre de 54 años muere por COVID-19. *Reporte Indigo*. Retrieved March 8, 2021, from https://www.reporteindigo.com/reporte/hombre-fallece-covid-19-despues-de-recorrer-siete-diferentes-hospitales/

20 CONNECTAS. (2020, August 21). Corrupción. La pandemia dispara sospechas sobre funcionarios en América Latina. Retrieved September 3, 2020, from https://www.lanacion.com.ar/politica/corrupcion-la-pandemia-dispara-sospechas-funcionarios-america-nid2424584; Gozzer, S. (2020, April 11). Cómo afecta a America Latina la pugna entre países por conseguir respiradores, ventiladores y mascarillas. *BBC News Mundo*. Retrieved from https://www.bbc.com/mundo/noticias-america-latina-52233577

21 For an examination of these issues see Mehrotra, S., Rahimian, H., Barah, M., Luo, F., & Schantz, K. (2020). A model of supply-chain decisions for resource sharing with an application to ventilator allocation to combat COVID-19. *Naval Research Logistics (NRL)*. https://doi.org/10.1002/nav.21905;Pugh, J., Wilkinson, D., Palacios-González, C., & Savulescu, J. (2020). Beyond Individual Triage: The Regional Allocation of Life-Saving Resources Such As Ventilators in Public Health Emergencies. *Health Care Analysis. Forthcoming*.

22 Savulescu, J., Vergano, M., Craxì, L., & Wilkinson, D. (2020). An ethical algorithm for rationing life-sustaining treatment during the COVID-19 pandemic. *British Journal of Anaesthesia*. https://doi.org/10.1016/j.bja.2020.05.028

23 Parfit, D. (1978). Innumerate Ethics. *Philosophy & Public Affairs. 7*(4), 285-301.

24 Kamm, F.M. (2007). Nonconsequentialism. *Intricate Ethics: Rights, Responsibilities, and Permissible Harm*. Oxford; New York: Oxford University Press: 11-48.

25 Bagenstos, S.R. (2020). Who Gets the Ventilator? Disability Discrimination in COVID-19 Medical-Rationing Protocols. *The Yale Law Journal*. (130), 1-25. Savulescu, Cameron, Wilkinson (op. cit. note 8)

26 This position is shared by, for example, Wasserman, D., Persad, G., & Millum, J. (2020). Setting priorities fairly in response to Covid-19: identifying overlapping consensus and reasonable disagreement. *Journal of Law and the Biosciences. 7*(lsaaO44). https://doi.org/10.1093/jlb/lsaa044

27 It bears noticing that some, like Samuel Bagenstos, reject this position. Bagenstos (op. cit. note 25) : 1–25 They maintain, for example, that it is discriminatory to deny ventilators to patients who will require prolonged support (because they have an underlying disability or chronic illness) in order to prioritise other patients who will need them only for a short period of treatment. Savulescu et al. have addressed this issue and argued that during the pandemic we should save the greatest numbers; Savulescu, Vergano, Craxì, Wilkinson (op. cit. note 22); Savulescu, Cameron, Wilkinson (op. cit. note 8)

28 Reporte Indigo (op. cit. note 20)

29 See Pugh, Wilkinson, Palacios-González, Savulescu (op. cit. note 21) for how the population density principle would work under egalitarian considerations.

30 The basic reproductive number (R_0_) is the expected number of cases directly generated by one case where all the population is susceptible to infection. This number does not depend solely on biological facts, but it “is an estimate of contagiousness that is a function of human behavior and biological characteristics of pathogens”. Delamater, P.L., Street, E.J., Leslie, T.F., Yang, Y.T., £Jacobsen, K.H. (2019). Complexity of the Basic Reproduction Number (R0). *Emerging Infectious Diseases. 25*(1), 1-4. https://doi.org/10.3201/eid2501.171901

31 Broome, J. (1984). Selecting people randomly. *Ethics. 95*(1), 38-55.

32 DePillis, L., Spies, M., Kaplan, J., Edwards, K., & Chen, C. (2020, March 20). Here’s Why Florida GotAII the Emergency Medical Supplies It Requested While Other States Did Not. Retrieved August 18, 2020, from https://www.propublica.org/article/heres-why-florida-got-all-the-emergency-medical-supplies-it-requested-while-other-states-did-not

33 Spector-Bagdady, K., Laventhal, N., Applewhite, M., Firn, J.I., Hogikyan, N.D., Jagsi, R.,... Shuman, A.G. (2020). Flattening the Rationing Curve: The Need for ExplicitGuidelines for Implicit Rationing during the COVID-19 Pandemic. *The American Journal of Bioethics: AJOB. 20*(7), 77-80. https://doi.org/10.1080/15265161.2020.1779409

34 Fergusson, N.A., Ahkioon, S., Nagarajan, M., Park, E., Ding, Y., Ayas, N Griesdale, D.E.G. (2020). Association of intensive care unit occupancy during admission and inpatient mortality: a retrospective cohort study. *Canadian Journal of Anesthesia/Journal Canadien d’anesthésie. 67*(2), 213-224. https://doi.org/10.1007/sl2630-019-01476-8; Gershengorn, H.B., Harrison, D.A., Garland, A., Wilcox, M.E., Rowan, K.M., & Wunsch, H. (2017). Association of Intensive Care Unit Patient-to-lntensivist Ratios With Hospital Mortality. *JAMA Internal Medicine*. *177*(3), 388-396. https://doi.org/10.1001/jamainternmed.2016.8457; Viglianti, E.M., & Iwashyna, T.J. (2017). Toward the Ideal Ratio of Patients to Intensivists: Finding a Reasonable Balance. *JAMA Internal Medicine. 177(3)*, 396-398. https://doi.org/10.1001/jamainternmed.2016.8476: Sakr, Y., Moreira, C.L., Rhodes, A., Ferguson, N.D., Kleinpell, R., Pickkers, P.,... Investigators, on behalf of the E.P. of I. in I.C.S. (2015). The Impact of Hospital and ICU Organizational Factors on Outcome in Critically III Patients: Results From the Extended Prevalence of Infection in Intensive Care Study*. *Critical Care Medicine. 43*(3), 519-526. https://doi.org/10.1097/CCM.0000000000000754

35 Even when here we only mention ICU personnel it must be clear that we mean to capture all personnel that have the skills for adequately treating these patients.

36 Staff Forbes. (2020, September 3). México, el país con más personal médico muerto porCovid-19: reporteAI. Retrieved Februaryll, 2021, from https://www.forbes.com.mx/noticias-mexico-pais-con-mas-personal-medico-muerto-por-coronavirus/

37 This would not happen if we opened, for example, a new (field) hospital with ICU beds. The most common obstacles for doing so relate to staffing the hospital and obtaining all the other necessary resources for providing adequate care.

38 Alternatively, there may be physical limits to the numbers of additional ventilators that can be accommodated, for example due to a lack of oxygen outlets, or a maximum flow rate of oxygen that can be delivered.

39 Reliable mortality statistics for COVID-19 are necessarily delayed, since patients often require prolonged intensive care stays, and may die (or survive) after a long period of hospitalisation.

40 Mortality statistics in some regions have been reported to be low (e.g. Mexico, Brazil, Venezuela, Russia), however, the accuracy of these figures have been disputed. There may be political reasons to report a lower mortality rate. Furthermore, if the distribution of ventilators is known to be linked to mortality rates, there would be an incentive for hospitals to report lower mortality figures.

41 Adam, S., Castillo, M., & Barriguete, A. (2020, May 18). Las actas sobre el número real de muertos con Covid-19 en CDMX. Retrieved August 18, 2020, from https://contralacorrupcion.mx/muertes-coronavirus-cdmx/; Ahmed, A. (2020, May 8). Cifras ocultas: México desatiende ola de muertes en la capital. *The New York Times*. Retrieved March 8, 2021, from https://www.nytimes.com/es/2020/05/08/espanol/america-latina/mexico-coronavirus.html

42 Lee, P.-I., Hu, Y.-L., Chen, P.-Y., Huang, Y.-C., & Hsueh, P.-R. (2020). Are children less susceptible to COVID-19? *Journal of Microbiology, Immunology, and Infection*. https://doi.org/10.1016/j.jmii.2020.02.011; Ruan, Q., Yang, K., Wang, W., Jiang, L., & Song, J. (2020). Clinical predictors of mortality due to COVID-19 based on an analysis of data of 150 patients from Wuhan, China. *Intensive Care Medicine. 46(5}*, 846-848. https://doi.org/10.1007/s00134-020-05991-x; Onder G, Rezza G, Brusaferro S. (2020). Case-Fatality Rate and Characteristics of Patients Dying in Relation to COVID-19 in Italy. *JAMA*, https://jamanetwork.com/journals/jama/fullarticle/2763 667

43 It would also, potentially, save the most life years, since younger patients with COVID-19 will likely live for longer on average than older patients.

44 Other ethical considerations, which we are not considering in this paper, might lead to the opposite conclusion. For example, consideration of saving the most life years (and not simply the most lives), could favour directing ventilators to a younger higher risk population.

45 Savulescu, Cameron, Wilkinson (op. cit. note 8)

46 Pandey, V. (2020, May 19). Huge crowds as lockdown sparks mass migration. *BBC News*. Retrieved March 8, 2021, from https://www.bbc.co.uk/news/av/world-asia-india-52093574

47 Savulescu, J., & Cameron, J. (2020). Why lockdown of the elderly is not ageist and why levelling down equality is wrong. *Journal of Medical Ethics. 46*(11), 717-721. https://doi.org/10.1136/medethics-2020-106336

48 INFOBAE. (2020, June 16). Coronavirus en México: contagios de COVID-19 sedisparan en niñas, niños y adolescentes hasta en un 313%. Retrieved September4, 2020, from /america/mexico/2020/06/16/coronavirus-en-mexico-contagios-de-covid-19-se-disparan-en-ninas-ninos-y-adolescentes-hasta-en-un-313/

49 Forbes Staff. (2020, June 14). Las comunidades indígenas presentan mayor letalidad porCovid-19 en México. Retrieved September 4, 2020, from https://www.forbes.com.mx/actualidad-las-comunidades-indigenas-presentan-mayor-letalidad-por-covid-19-en-mexico/

50 Vega, M. (2016, February 26). I⋂díge⋂as y salud: Aquí no hay clínica, ni médico, ni medicinas... Retrieved September4, 2020, from http://www.animalpolitico.com/indigenasysalud/aqui-no-hay-medico-ni-clinica-ni-medicinas.html

51 For discussion of such a strategy in the context of vaccine rationing, see Schmidt, H. (2020). Vaccine Rationing and the Urgency of Social Justice in the Covid-19 Response. *Hastings Center Report. 50(3)*, 46-49. https://doi.org/10.1002/hast.1113

52 The number of ICU patients that physicians and nurses can look over will vary according to each jurisdiction.

